# Fucoxanthin from *Undaria pinnatifida*: Photostability and Coextractive Effects

**DOI:** 10.3390/molecules18066298

**Published:** 2013-05-29

**Authors:** Anna Piovan, Roberta Seraglia, Bruno Bresin, Rosy Caniato, Raffaella Filippini

**Affiliations:** 1 Department of Pharmaceutical and Pharmacological Sciences, University of Padova, Via Marzolo 5, Padova 35131, Italy; E-Mails: rosy.caniato@unipd.it (R.C.); raffaella.filippini@unipd.it (R.F.); 2 CNR-ISTM, Corso Stati Uniti 4, Padova 35100, Italy; E-Mail: roberta.seraglia@adr.pd.cnr.it; 3 ARPA-FVG Regional Agency for Environmental of Friuli Venezia Giulia Region, Via delle Acque 28, Pordenone 33170, Italy; E-Mail: bdbres@alice.it

**Keywords:** fucoxanthin, *Undaria*, photostability, coextractives, QuEChERS

## Abstract

Fucoxanthin is one of the most abundant carotenoids and possesses a number of beneficial medicinal qualities which include its anti-oxidant, anti-obesity and anti-cancer properties. In this study, the photostability of fucoxanthin in extracts with different chemical profiles was studied. The extracts were obtained from *Undaria pinnatifida*, a seaweed rich in this carotenoid, using conventional liquid solvent extraction procedures and the QuEChERS method. All the extracts contained all-*trans*-fucoxanthin as the major compound. Conventional procedures produced a fucoxanthin purity of lower than 50%, whereas after liquid-liquid partition, PSA cleanup, and PSA and GCB cleanup (QuEChERS method) fucoxanthin purity increased to 70%, 86%, and 94%, respectively. Although in the acetone extract the initial content of fucoxanthin was the highest, results demonstrate that coextractives play an important role in enhancing the rate of photodegradation. After light exposure, the conventional extracts lost around 90% of the initial fucoxanthin content. On the other hand, the extracts obtained by the QuEChERS method showed significantly higher light stability than the conventional extracts. These results suggest that the QuEChERS method could be used and further improved to obtain more purified and stable extracts for fucoxanthin from *U. pinnatifida*.

## 1. Introduction

**Figure 1:**
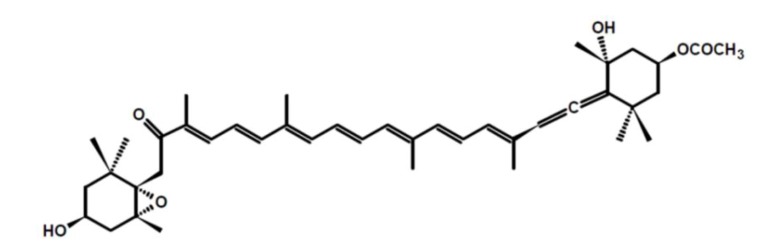
Fucoxanthin.

Fucoxanthin is one of the most abundant carotenoids, and contributes to more than 10% of the estimated total production of carotenoids in Nature, especially in the marine environment [1]. Fucoxanthin is a pigment, along with chlorophylls and β-carotene, widely distributed in brown algae and diatoms [2]. It has an unusual structure with an allenic bond and 5,6-monoepoxide in its molecule (Figure 1).

*Undaria pinnatifida*, a brown seaweed better known as wakame, is a rich source of fucoxanthin [3]. Actually, *U. pinnatifida* is widely used as a human food in many countries especially Korea and Japan, and it is becoming increasingly popular in the European market, above all in the form of extracts used as food supplements. The numerous activities attributed to *U. pinnatifida* products are essentially linked to their fucoxanthin content [4]. There is indeed a growing evidence from *in vitro* and *in vivo* experiments suggesting that fucoxanthin has health promoting effects because of its strong anti-oxidant properties [5,6,7,8]. Fucoxanthin provides protective effects on liver, blood vessels of the brain, bones, skin and eyes. It has anti-obesity and anti-diabetic properties, and anti-inflammatory and anti-malarian effects. Moreover it is very effective in inhibiting cell growth and inducing apoptosis in human cancer cells. Particularly, the anti-adult T-cell leukemia effects, the induction of apoptosis in human leukemia cells, and the anti-obesitive effect of fucoxanthin are distinctly more potent than that of β-carotene and astaxanthin. The unique effects of fucoxanthin is due its characteristic chemical structure, and carotenoids without an allenic bond are not active [4,9,10]. Recently, it has also been demonstrated that pretreatment with fucoxanthin improves the chemotherapeutic efficacy of cisplatin by enhancing the inhibition of cell proliferation of human hepatoma HepG2. These results suggest that the combined treatment of fucoxanthin and cisplatin may provide a novel therapeutic approach to decrease cisplatin-induced drug resistance [11,12].

Owing to these properties fucoxanthin has attracted considerable interest both as nutraceuticals and pharmaceuticals, and there is an increasing interest in the effects of this seaweed carotenoid as a functional supplement in human diets helping to enhance the nutritional profiles of foods such as pasta, beverages, cakes and spreads [13,14,15,16].

Though chemical synthesis of fucoxanthin is possible, it is very expensive and therefore the viability of obtaining directly from brown seaweeds should not be overlooked [4]. Indeed there have been several studies which have focused on its extraction and purification from seaweeds. A crude oil, a mixture of carotenoids and polyphenols, was obtained from *U. pinnatifida* using supercritical carbon dioxide [17] and a method for its separation and purification from edible brown algae by microwave-assisted extraction coupled with high-speed countercurrent chromatography has been developed [18]. However, the most common way for fucoxanthin extraction is by liquid solvent extraction whereas the available commercial products are mainly constituted of extracts [19].

Fucoxanthin is highly susceptible to degradation and this can lead to *cis-trans* isomerisation, oxidative cleavage and/or epoxidation of the backbone [20]. While the deleterious effects of heat, light, or coextractives on fucoxanthin have regularly been cited for almost half a century, the extent of the impact of these factors has rarely been determined [21,22,23]. In this work, we have studied the effects of coextractives present in different extracts on the photostability of the fucoxanthin. 

## 2. Results and Discussion

Frequent references are made in literature concerning procedures that impact on fucoxanthin stability [24,25,26]; however, the extent of these effects have not as yet been quantified. In this study, the photostability of the fucoxanthin in *Undaria pinnatifida* extracts with different chemical profiles was studied.

We used fresh starting material and not exhaustive extraction procedures in order to prevent as far as possible any degradation event occurring during the sample processing steps. The extractions were performed by using three different solvents (methanol, acetone, acetonitrile) with different extraction power both *versus* fucoxanthin and other compounds present in *U. pinnatifida*. The extracts were obtained in an ultrasonic bath (conventional procedures) and with the Quick, Easy, Cheap, Effective, Rugged, and Safe (QuEChERS) method [27]. The QuEChERS method is the most commonly applied prep method for the determination of pesticide residues from a variety of fruit and vegetables, fatty food matrixes like milk and eggs, and water [28]. This method involves an extraction with acetonitrile partitioned from the aqueous matrix using anhydrous magnesium sulphate (MgSO_4_) and sodium chloride (NaCl) (acetonitrile raw extract) followed by a dSPE cleanup with MgSO_4_ and primary secondary amine (PSA) (cleanup) or a combination of PSA and graphitized carbon black (GCB) (additional cleanup). The use of the QuEChERS method allowed us to evaluate the fucoxanthin photodegradation of increasingly purified acetonitrile extracts.

The identification of fucoxanthin in the different extracts was confirmed with an external calibration *i.e.*, by comparing retention time, and UV-Vis and MS/MS spectra, respectively, of the samples to those of the standard. The quantitative analyses were performed by HPLC UV-Vis. The validation data of the method are shown in Table 1.

**Table 1:** Regression curve data, detection limit, quantification limit and reproducibility.

Regression curve data	Detection limit	Quantification limit	Reproducibility (%RSD)	
y = 903598x + 26548	*(µg/mL)*	*(µg/mL)*	*intra-day*	*inter-day*
r^2^ = 0.9999	0.0112	0.035	<4	<7

**Figure 2:**
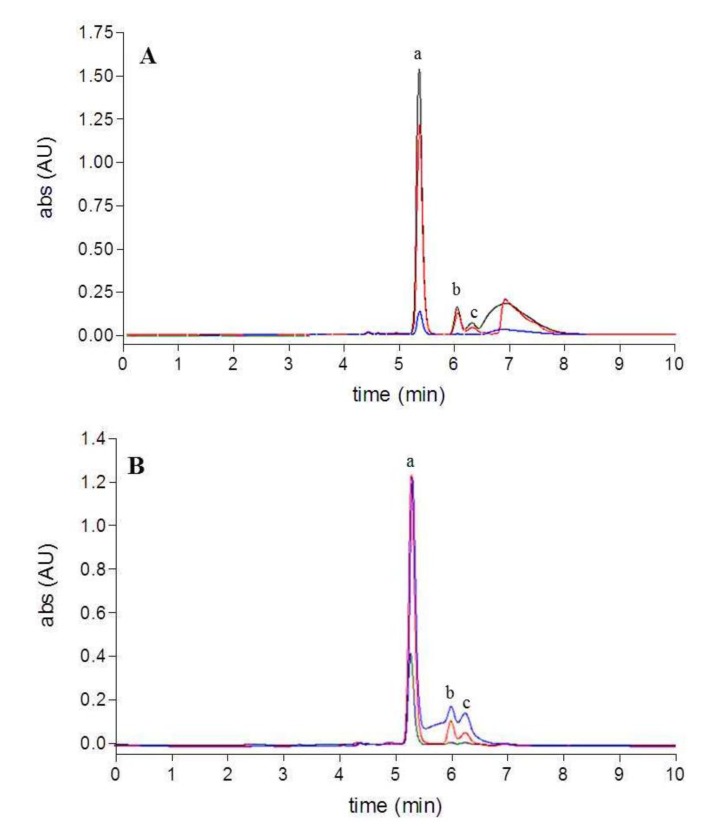
HPLC chromatograms (449 nm): (**A**) conventional extracts (acetone-black, acetonitrile-red, methanol-blue); (**B**) extracts obtained using QuEChERS method (acetonitrile raw extract-blue, PSA cleanup-red, GCB additional cleanup-black).

The use of the QuEChERS method resulted in a visible cleanup of the extracts compared to the conventional procedures showing differences in color. The HPLC chromatograms of the extracts are shown in Figure 2. All the extracts contained all-*trans*-fucoxanthin as the major compound (RT = 5.5 min, a) and two other minor peaks (RT = 5.5–6 min, b and c) around the fucoxanthin peak, which showed similar UV-Vis spectra to fucoxanthin, as reported by Fung *et al.* [29]. These minor peaks were identified by MS/MS spectra as the *cis*-isomers of fucoxanthin. A broad and tailing peak was detected in all the conventional extracts and assigned to other coeluted matrix components; this peak was not present in the extracts obtained with the QuEChERS approach. It is worth noting that the acetonitrile raw extract (QuEChERS method) was obtained from an acetonitrile aqueous solution by salt-induced phase-separation. The different polarity of the solvent in presence of salts gave a greater matrix cleanup of the extract than with the conventional procedure with acetonitrile. The impurity content was determined as a percentage of the total area of all the peaks. The conventional procedures allowed for fucoxanthin purity lower than 50%, whereas in the acetonitrile raw extract (liquid-liquid partition), after PSA cleanup and improved cleanup (QuEChERS approach) fucoxanthin purity reached 70%, 86%, and 94% respectively.

Table 2 shows the fucoxanthin contents in the different extracts. Considering the QuEChERS method, the fucoxanthin amounts in the acetonitrile raw extract and after PSA cleanup were not significantly different (using Tukey’s multiple range test, *p* < 0.05). Applying the improve cleanup with PSA and GCB, GCB had a significant effect on the recovery of fucoxanthin and drove to its drastic lost: only one third of the fucoxanthin was recovered with GCB compared to the acetonitrile raw extract. 

**Table 2:** Fucoxanthin contents (µg/mL) in the conventional extracts (acetone, acetonitrile, methanol) and in the extracts obtained with the QuEChERS method (acetonitrile raw extract, PSA cleanup, GCB additional cleanup).

Conventional procedures				QuEChERS method		
acetone	acetonitrile	methanol		MeCN raw extract	PSA cleanup	GCB additional cleanup
12.3 ± 1.10 ^e^	9.8 ± 0.99 ^c^	1.0 ± 0.12 ^a^		10.5 ± 0.98 ^c,d^	10.6 ± 1.01 ^d^	3.3 ± 0.29 ^b^

Means ± SD. Numbers followed by the same lowercase letter did not differ statistically (Tukey test, *p* > 0.05).

In order to evaluate the effects of coextractives on the stability of the fucoxanthin, all the extracts, fucoxanthin standard solutions and fucoxanthin standard solutions added of ascorbic acid were placed in direct daylight to compare the effects of coextractives and antioxidant ascorbic acid on fucoxanthin stability under light conditions.

**Figure 3:**
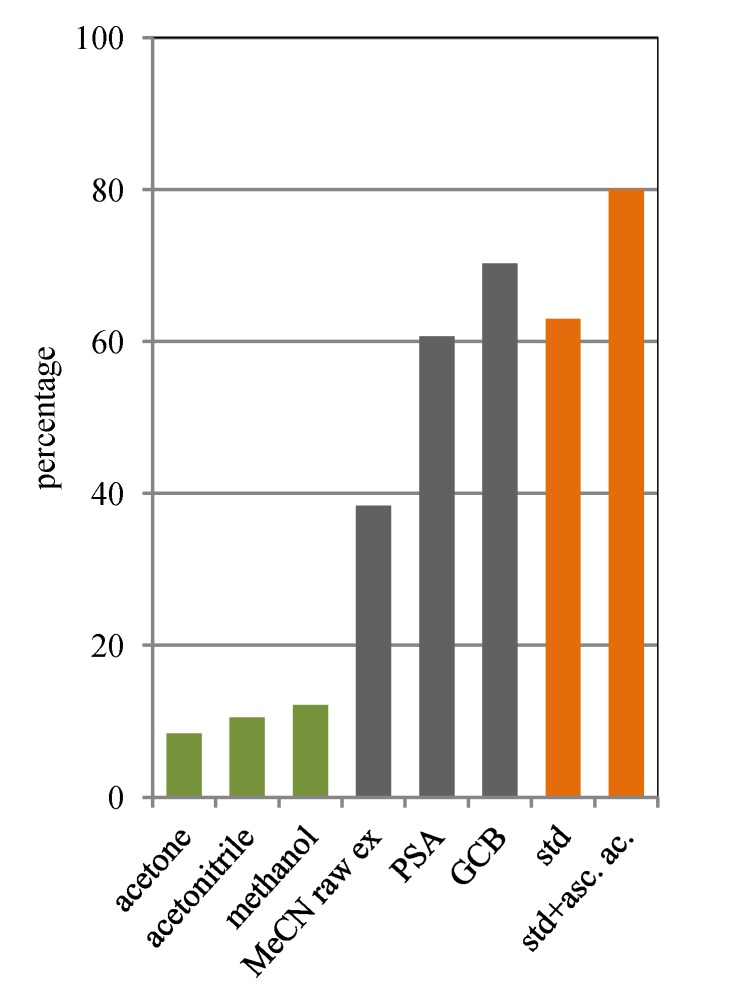
Fucoxanthin content after light exposure expressed as a percentage of the initial content in the conventional extracts (acetone, acetonitrile, methanol; green), in the extracts obtained with the QuEChERS method (acetonitrile raw extract, PSA, GCB; grey) and in standard solutions (with and without ascorbic acid; orange).

Figure 3 shows the fucoxanthin content as percentage of the initial concentration after light exposure. Results here indicate that fucoxanthin is susceptible to photodegradation. Although Mise *et al.* [30] reported that pure fucoxanthin is unstable but, the fucoxanthin extracted from the alga is rendered stable by the coexisting antioxidants, our data clearly demonstrate that conventional extracts are the least stable, which could be related to the matrix effects. Indeed all the solutions had lost around 90% of their initial fucoxanthin contents. In the acetonitrile raw extract (QuEChERS method) the content of fucoxanthin was reduced to 40% of its initial concentration. A steady and significant increase of the stability was observed after PSA cleanup and additional cleanup (PSA and GCB). The extract obtained after PSA cleanup retained around 60% of the initial concentration; in the extract obtained applying the additional cleanup the fucoxanthin was 70% of the initial concentration. The fucoxanthin standard solution and the fucoxanthin standard solution added of ascorbic acid retained around 60% and 80% of the initial concentration respectively.

**Figure 4:**
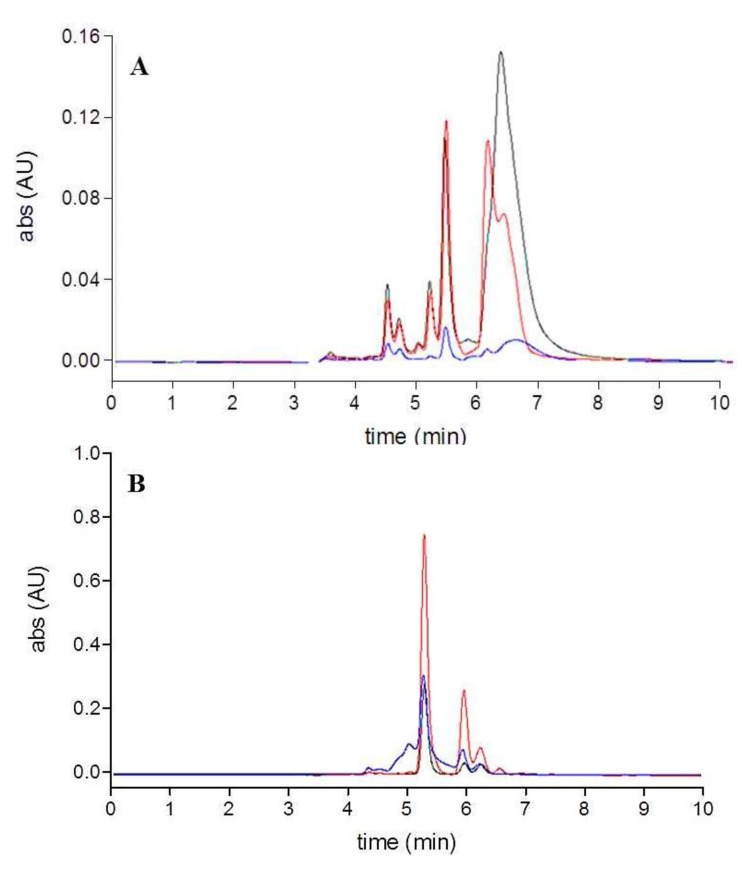
HPLC chromatograms (449 nm) obtained after light exposure of the extracts: (**A**) conventional extracts (acetone-black, acetonitrile-red, methanol-blue); (**B**) extracts obtained using QuEChERS method (acetonitrile raw extract-blue, PSA cleanup-red, GCB additional cleanup-black.

**Figure 5:**
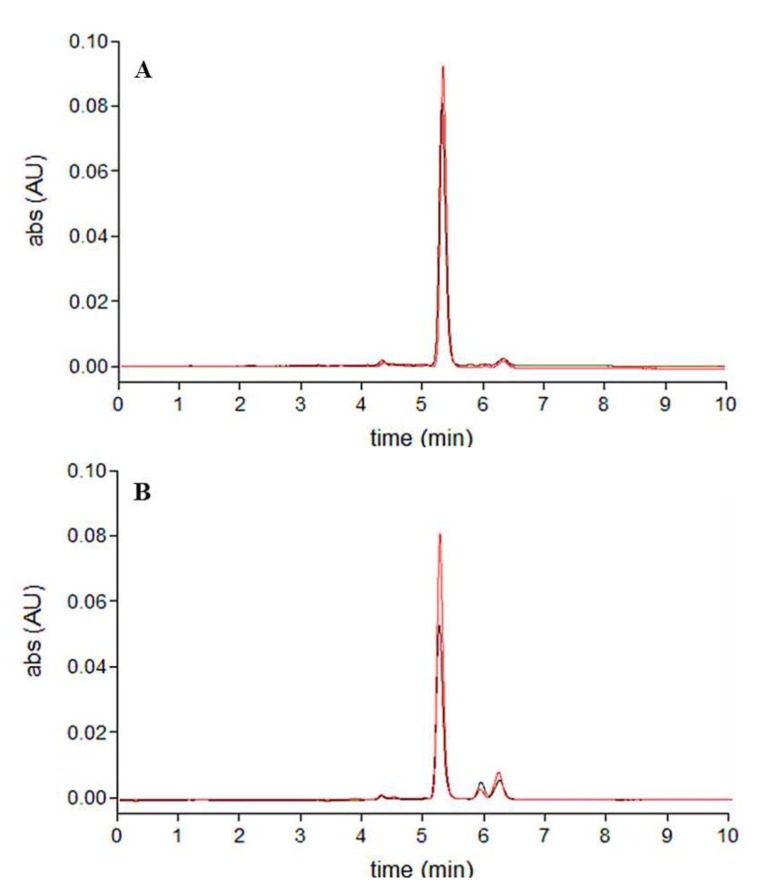
HPLC chromatograms (449 nm) of fucoxanthin standard solutions: (**A**) fucoxanthin standard solution-black, fucoxanthin standard solution with ascorbic acid-red; (**B**) after light exposure.

As shown by the HPLC chromatograms (Figure 4 and Figure 5), the degradation pattern of fucoxanthin in the conventional extracts on the one hand, in the extracts obtained with the QuEChERS method and in standard solutions on the other hand was notably different. Light exposure of fucoxanthin standard solutions and extracts obtained with the QuEChERS method predominantly leads to the formation of *cis*-isomers, whereas different unidentified compounds, displaying a retention time between 4–5 and 5.5–8 min, are mainly formed in the conventional extracts. Again, the observed fucoxanthin loss was not mirrored by the appearance of comparable levels of isomers or degradation products as already reported [22].

Fucoxanthin is subject to isomerization and oxidation which are recognized as important reactions causing carotenoid degradation [20]. The different pattern and rate of fucoxanthin degradation occurring in the conventional extracts most probably depend on other components present in solution able to catalyze the oxidation of fucoxanthin, producing different products.

The results suggest that the first stage of degradation is the isomerization of all-*trans*- to more oxidable *cis*-isomers. Indeed in the presence of ascorbic acid, standard fucoxanthin was subject to isomerization, but the further oxidation was significantly prevented by the antioxidant agent. 

**Figure 6:**
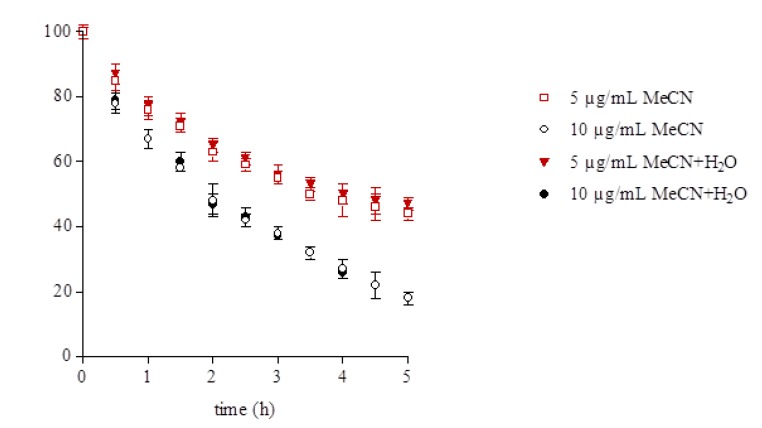
Fucoxanthin content after light exposure expressed as percentage of the initial content in standard solutions (5 and 10 µg/mL) in pure acetonitrile solvent (MeCN) and in acetonitrile/water mixture (1:1, v/v) (MeCN + H_2_O).

On the basis of these results, a second set of experiments was carried out in order to evaluate a possible role of the water present in the conventional extracts and in the raw extract on the degradation rate. Moreover the fucoxanthin degradation rate in relation to its concentration was evaluated (Figure 6). No differences were observed in the degradation rate in pure acetonitrile solvent and in the acetonitrile/water mixture. On the contrary the results show that the degradation is faster at the higher fucoxanthin concentration. Indeed after only 30 min of light exposure the 10 µg/mL solutions had lost 20% of their initial content *i.e.*, around 2 µg/mL, whereas the 5 µg/mL solutions had lost 15% of their initial content *i.e.*, around 0.75 µg/mL. The contents of fucoxanthin after 5 h exposure were reduced by 80% and 55% respectively to around 2 µg/mL in all the solutions. 

These results support the statement that the observed different pattern and rate of fucoxanthin degradation in the extracts depend on coextractives present in solution rather than the presence of water or fucoxanthin concentration. Indeed the extracts obtained by conventional procedures with acetone and acetonitrile, and the acetonitrile raw extract and the extract obtained by PSA cleanup (QuEChERS method) had a similar initial fucoxanthin content but underwent different rates of degradation. Moreover, the extract obtained after additional cleanup (PSA and GCB) (3 µg/mL) having a fucoxanthin purity of 94% showed a degradation rate very similar to the standard solutions (5 µg/mL).

Table 3 shows the fucoxanthin content in the different extracts after light exposure. It needs to be pointed out that, although in the acetone extract (conventional procedure) the initial content of fucoxanthin was the highest (12.3 µg/mL), the coextractives played an important role in enhancing the rate of photodegradation. After only 90 min of light exposure, the content of fucoxanthin was reduced by 90% to 1.0 µg/mL. On the other hand, even if the extract obtained after PSA cleanup (QuEChERS method) had a lower fucoxanthin content (10.6 µg/mL), the extract showed significantly increased light stability since the fucoxanthin content after light exposure was decreased by 40% to 6.4 µg/mL. As pointed out above, there was no significant difference between the fucoxanthin degradation rate in the extract obtained after PSA cleanup and in standard solution, and the fucoxanthin content after light exposure was around 60% of the initial concentrations in both the samples.

**Table 3:** Fucoxanthin content (µg/mL) in the conventional extracts (acetone, acetonitrile, methanol) and in the extracts obtained with the QuEChERS method (acetonitrile raw extract, PSA cleanup, GCB additional cleanup) after light exposure.

Conventional procedures				QuEChERS method		
acetone	acetonitrile	methanol		MeCN raw extract	PSA cleanup	GCB additional cleanup
1.0 ± 0.07 ^b^	1.0 ± 0.08 ^b^	0.1 ± 0.01 ^a^		4.0 ± 0.30 ^d^	6.4 ± 0.57 ^e^	2.3 ± 0.18 ^c^

Means ± SD. Numbers followed by the same lowercase letter did not differ statistically (Tukey test, *p* > 0.05).

## 3. Experimental

### 3.1. Materials

Solvents: methanol was purchased from Merck; acetonitrile and acetone from Fluka. Water was purified with a Milli-Q deionization unit (Millipore). All-*trans*-fucoxanthin with ≥95% purity was purchased from Sigma-Aldrich.

QuEChERS materials were obtained from commercial suppliers. For the initial extraction step, QuEChERS Extraction SPE Kits (Agilent, Santa Clara, CA, USA) were used consisting of 50 mL plastic centrifuge tubes containing 4 g anhydrous magnesium sulphate (MgSO_4_) and 1 g sodium chloride (NaCl). For cleanup Q-sep™ QuEChERS dSPE Tubes for Extract Clean-Up (Restek) were used consisting of 2 mL mini-centrifuge tubes containing 150 mg anhydrous MgSO_4_, 25 mg primary secondary amine (PSA) sorbent, with and without 7.5 mg graphitized carbon black (GCB).

*Undaria pinnatifida* samples were harvested from the lagoon of Venice (Italy) in June 2012.

### 3.2. Standard Stock Solution

A stock solution of fucoxanthin was prepared in acetonitrile at a concentration of 1 mg/mL and stored at −20 °C in amber-colored vials to protect fucoxanthin from light. The working solutions were accurately diluted with acetonitrile just prior to use.

### 3.3. Sample Preparation

The collected seaweed samples were washed with water three times. The cleaned samples were stored at −20 °C until use. Brown seaweed samples (about 500 g) made up of whole algae were homogenised with an Ultraturrax (Janke & Kunkel IKA Labortechnik, Staufen, Germany). Extractions were performed using conventional procedures and the QuEChERS approach.

Conventional procedures: homogenised seaweed samples (10 g) were extracted with acetonitrile, methanol and acetone (10 mL) in an ultrasonic bath at room temperature (10 min).

QuEChERS approach: the original method entailed the following steps: (a) weigh 10 g of thoroughly homogenized sample into a 50 mL centrifuge tube; (b) add 10 mL acetonitrile; (c) add 4 g anhydrous MgSO_4_ and 1 g NaCl; shake vigorously for 1 min by hand; (d) centrifuge the tube at 3000 rpm for 1 min; (e) transfer 1 mL of the upper organic phase (raw extract) to a mini-centrifuge tube and subject it to a dispersive cleanup by mixing it with 150 mg anhydrous MgSO_4_, 25 mg PSA; shake by hand for 30 s; (f) centrifuge the tube at 3,000 rpm for 5 min; transfer the final extract in a vial for analysis. A first set of extracts was prepared according to the original QuEChERS method; in a second set dSPE was performed using a combination of PSA and GCB (additional cleanup).

The extractions were performed in triplicates. The extracts were conveniently diluted before the analyses.

### 3.4. Light Exposure Experiments

In a first set of experiments, freshly prepared 1 mL aliquots of extracts, and standard fucoxanthin solutions (10 µg/mL) with and without ascorbic acid (equimolar concentration) were placed in clear glass sample vials at room temperature in direct daylight (2,500 lux; 90 min).

In a second set of experiments standard fucoxanthin solutions (5 and 10 µg/mL) in pure acetonitrile solvent and in acetonitrile/water mixture (1:1, v/v) were placed in clear glass sample vials at room temperature in direct daylight (2,500 lux). Samples were collected at 30 min intervals for 5 h. The experiments were performed in triplicates.

### 3.5. HPLC UV-Vis Analysis

#### 3.5.1. Methodology

HPLC analysis was performed on ChromQuest (Thermoseparation, San Jose, CA, USA) pump P4000 equipped with a photodiode array detector UV6000. The data were recorded and processed using ChromQuest Chromathography Workstation. The separation was achieved with a reversed-phase analytical column, Gemini C6-Phenyl column (250 × 4, 60 mm i.d., 5 µm; Phenomenex, Torrance, CA, USA), using an isocratic elution, and the injection volume was 20 µL. The mobile phase was composed of methanol:water, 90:10; the flow rate was 1 mL/min. UV-Vis spectra were recorded in the 200–700 nm range; chromatograms were acquired at 449, 330 and 254 nm.

#### 3.5.2. Method Validation

The linearity of the method was evaluated by using six calibration standard solutions over a range of 0.05–7 µg/mL. The calibration curve was established by plotting peak area ratios of calibration solutions *vs* the nominal concentrations of fucoxanthin. The linearity was determined using linear regression analysis. The limit of detection was defined as the lowest concentration level resulting in a peak area of three times the baseline noise (S/N > 3) in solvent. The limit of quantification was defined as the lowest concentration level resulting in a peak area of ten times the baseline noise (S/N > 10) in solvent. Measurements of the intra- and inter-day variability were utilised to determine the reproducibility of the method. Fucoxanthin standard solutions were analysed to determine the intra-day repeatability (examined in one day) and inter-day repeatability (determined on 3 different days). The relative standard deviation (RSD) was calculated as a measurement of method reproducibility (*n* = 3). 

### 3.6. HPLC MS/MS Analysis

The HPLC conditions were those already described. A post-column split was employed to deliver approximately 250 µL/min to the electrospray interface. The ESI-MS analysis was performed using a LCQ Deca Ion Trap (Thermo Finnigan, San Jose, CA, USA), operating in the positive ion mode. Sheath gas and auxiliary gas flow rates were 60 and 40 (arbitrary units) respectively, spray voltage 4 kV and entrance capillary temperature 280 °C. Collision-induced dissociation spectra were obtained applying a supplementary RF voltage, 6 V, to the end caps of the ion trap. The data were recorded and processed using Xcalibur Workstation.

### 3.7. Statistical Analysis

Data were subjected to analysis of variance (ANOVA) to assess significant differences between the fucoxanthin content in the different extracts. Significance between the mean values was tested by Tukey's test at the confidence level of *p* ≤ 0.05.

## 4. Conclusions

In this work it was demonstrated that coextractives play an important role in enhancing the rate of fucoxanthin photodegradation. After light exposure, the conventional extracts lost around 90% of their initial fucoxanthin concentrations, whereas there was no significant difference between the fucoxanthin degradation rate in the extract obtained after PSA cleanup (QuEChERS method) and in standard solution with 40% loss of fucoxanthin. To the best of our knowledge, this is the first report on the application of the QuEChERS method to obtain fucoxanthin extracts from *U. pinnatifida*. Compared to conventional extraction techniques, the QuEChERS method provided a progressive extract cleanup and extract stability.

The promising results of fucoxanthin activity studies prompt the development of extraction and purification methods to obtain pure fucoxanthin. The above results suggest that the QuEChERS method could be used and further improved to obtain purified and stable extracts for fucoxanthin from *U. pinnatifida*.
